# Lipoprotein(a): Cellular Effects and Molecular Mechanisms

**DOI:** 10.1155/2012/923289

**Published:** 2012-09-06

**Authors:** Kirsten Riches, Karen E. Porter

**Affiliations:** Division of Cardiovascular Medicine, Leeds Institute of Genetics, Health and Therapeutics (LIGHT) and Multidisciplinary Cardiovascular Research Centre (MCRC), University of Leeds, Leeds LS2 9JT, UK

## Abstract

Lipoprotein(a) (Lp(a)) is an independent risk factor for the development of cardiovascular disease (CVD). Indeed, individuals with plasma concentrations >20 mg/dL carry a 2-fold increased risk of developing CVD, accounting for *~*25% of the population. Circulating levels of Lp(a) are remarkably resistant to common lipid lowering therapies, and there are currently no robust treatments available for reduction of Lp(a) apart from plasma apheresis, which is costly and labour intensive. The Lp(a) molecule is composed of two parts, an LDL/apoB-100 core and a unique glycoprotein, apolipoprotein(a) (apo(a)), both of which can interact with components of the coagulation cascade, inflammatory pathways, and cells of the blood vessel wall (smooth muscle cells (SMC) and endothelial cells (EC)). Therefore, it is of key importance to determine the molecular pathways by which Lp(a) exerts its influence on the vascular system in order to design therapeutics to target its cellular effects. This paper will summarise the role of Lp(a) in modulating cell behaviour in all aspects of the vascular system including platelets, monocytes, SMC, and EC.

## 1. Introduction

Elevated plasma lipoprotein(a) (Lp(a)) is an independent risk factor for the development of cardiovascular disease (CVD) [1]. It is synthesised and secreted by the liver and comprises a lipid core of LDL cholesterol and apoB-100, surrounded by a unique glycoprotein apolipoprotein(a) (apo(a)) [2]. Apo(a) shares homology with plasminogen, containing multiple copies of kringle 4 (KIV), one copy of kringle 5 (KV) and an inactive protease domain. KIV is present in numerous forms; there are single copies of KIV type 1 and 3–10, but multiple copies of KIV type 2 which give rise to the large variation in Lp(a) size (reviewed in [2]). The species distribution of Lp(a) is limited to humans and old world monkeys (a distant homolog is present in hedgehogs) due to expression of the apo(a) gene. Therefore, it is likely that it is the apo(a) moiety of Lp(a) that confers its pathogenicity. Transgenic animals have been generated to express human apo(a), or human apo(a) and apoB-100 to aid the study of Lp(a) and in general these have confirmed the observation that Lp(a) is atherogenic regardless of species (reviewed in [3]). 

Although Lp(a) was discovered almost 50 years ago [4] and its influence as a cardiovascular risk factor has been known since the 1980s [5, 6], its true physiological function remains unknown. It is highly likely that it plays a role in mediation of wound healing, as immunohistochemical analysis of healing wounds stained positively for apo(a)/apoB during the infiltration of immune cells, production of granulation tissue, and initiation of revascularisation [7]. In addition, a recent proteomics study determined that many of the proteins associated with Lp(a) were involved with the wound healing response [8]. 

In the absence of a defined physiological role of Lp(a), its pathophysiological role is undoubtedly that of a prominent risk factor for the development of CVD. Circulating levels of Lp(a) are not significantly modified by traditional lipid-lowering therapies [3, 9], and so alternative approaches to target its adverse functions specifically are necessary and may be of therapeutic value. This paper will focus on the detrimental effects of Lp(a) in the cardiovascular system including the coagulation cascade, inflammatory pathways and modulation of smooth muscle (SMC), and endothelial cell (EC) behaviour within blood vessel walls.

## 2. Thrombosis 

Following injury to the vessel wall, platelets become activated and trigger thrombus formation. Fibrin cross-links and stabilises the clot; during resolution it is broken down by plasmin to minimise vessel occlusion. Lp(a) has been demonstrated to act as a prothrombotic factor, interfering with clot biology at multiple levels, as follows.

### 2.1. Platelet Aggregation

Platelets are activated by exposure to collagen on the surface of damaged blood vessels, leading to secretion of dense granules to activate further platelets in a positive feedback loop. Aggregation occurs via fibrinogen binding to integrin *α*_IIb_ *β*_3_ on the platelet surface and clot formation is initiated [10]. Lp(a) has been reported to affect platelet activation/aggregation induced by various agonists; however, there is currently no clear consensus on whether it potentiates or attenuates their effects. 

Evidence of Lp(a) influencing the initial activation of platelets is scarce, although both Lp(a) and apo(a) alone have been demonstrated to promote activation via thrombin-receptor-activated hexapeptide (TRAP) [11]. However, the ability of Lp(a) to directly affect platelet aggregation is much more contentious. Studies have shown that both Lp(a) and apo(a) alone enhanced aggregation in response to arachidonic acid and TRAP [11, 12], yet had no effect on aggregation induced by collagen or thrombin [12]. Lp(a) had previously been demonstrated to inhibit aggregation induced by low concentrations of collagen (4 *μ*g/mL) [13]; however, in that case the inhibitory effect was not observed when collagen concentrations were increased to 10 *μ*g/mL [12]. Aggregation in response to platelet activating factor (PAF) has also been reported to be inhibited by Lp(a) [14]. In the circulation, Lp(a) has been found associated with PAF-acetylhydrolase (PAF-AH) [15]; however, inhibition of platelet aggregation also occurred when PAF-AH was removed from Lp(a) [14], indicating a dual inhibitory effect of Lp(a) on the PAF system. It is likely that the conflicting effects of Lp(a) on platelet aggregation are dependent on both the concentration of aggregation factor, and interactions and interplay of either apo(a) and apoB-100/LDL. For example, inhibition of aggregation in response to PAF was reportedly much more potent when apo(a) was removed from the Lp(a) molecule [14]. 

The antiaggregatory effects of Lp(a) may be mediated via its interaction with integrin *α*_IIb_ *β*_3_. Integrin *α*_IIb_ *β*_3_ is normally bound by fibrinogen to promote platelet aggregation, yet apo(a) can displace fibrinogen from the receptor [16] thus inhibiting this process. In addition, functional effects of Lp(a) can be dependent on modifications of the Lp(a) molecule—platelet granule secretion was altered when Lp(a) was modified by lipid peroxidation products or acetylation [17]. It is clear that the interaction of Lp(a) with platelets is complex and involves a balance between Lp(a) subunit binding, protein modifications, and the factor stimulating platelet aggregation.

### 2.2. Tissue Factor Pathway

Tissue factor (TF) is a key early component of the coagulation system leading to activation of thrombin [18]. Treatment of monocytes with Lp(a) or recombinant apo(a) (r-apo(a)) caused a 2-fold increase in production and surface association of TF due to activation of integrin *α*_M_ *β*_2_ and the nuclear factor kappa B (NF*κ*B) signalling cascade [19]. However, activation of TF displays cell-type specificity as treatment of human umbilical vein endothelial cells (HUVEC) with Lp(a) did not affect TF expression [20]. It is known that platelets express TF, and since they are responsive to Lp(a) it would be of potential interest to investigate the effect on TF in this cell type.

In addition to promoting TF expression in monocytes, Lp(a) may augment thrombosis further by binding and inhibiting tissue factor pathway inhibitor (TFPI). Whilst usually exhibiting thrombolytic properties, TFPI is reportedly inhibited by nanomolar concentrations of Lp(a) through the apo(a) moiety. In addition, TFPI and apo(a) appear colocalised in SMC-rich intimal regions of human atherosclerotic plaques, suggestive of functional effects [21]. 

### 2.3. Impairment of Plasminogen Activation

**Figure 1:**
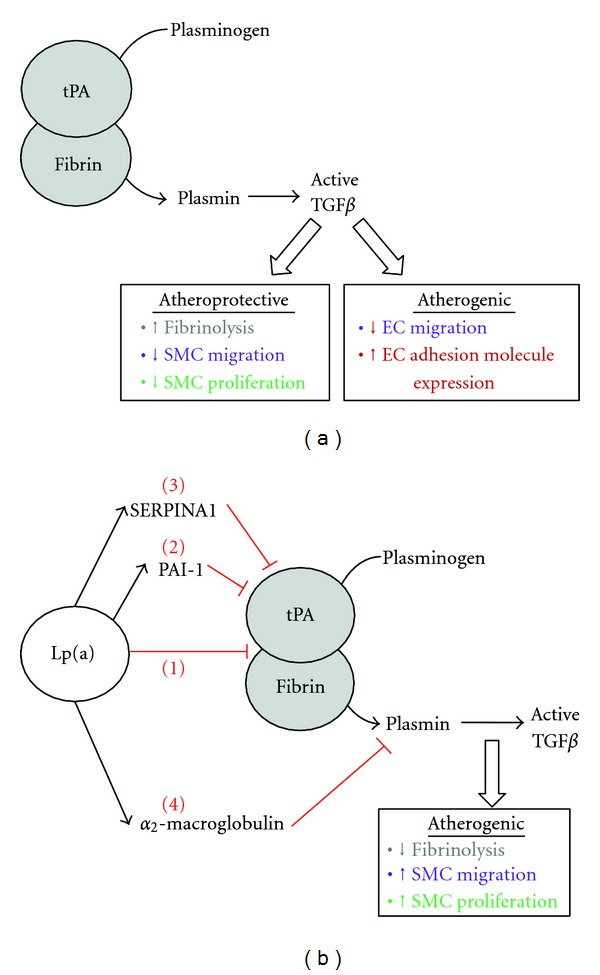
Prothrombotic actions of Lp(a). (a) Plasminogen is activated in a ternary complex comprising fibrin and tPA. Mature plasmin encourages thrombolysis and activates TGF*β*. TGF*β* has both atheroprotective effects (inhibition of SMC migration and proliferation) and atherogenic effects (inhibition of EC migration, induction of EC cell surface adhesion molecules). (b) Under conditions of elevated plasma Lp(a), plasminogen activation is impaired by multiple mechanisms: Lp(a) competes with plasminogen and tPA for fibrin binding (1). In addition, Lp(a) increases expression of PAI-1 (2) and associates with SERPIN1A (3), both leading to inhibition of tPA. Finally, Lp(a) can associate with *α*2-macroglobulin, a plasmin inhibitor (4). In combination, all these factors promote a prothrombotic environment.

Through its homology with plasminogen, Lp(a) can inhibit the formation of active plasmin. Plasminogen is activated extracellularly by a ternary complex comprising tissue plasminogen activator (tPA), plasminogen and fibrin. Active plasmin then dissociates from the complex and is able to activate TGF*β* and degrade fibrin within clots (Figure 1(a)). However, in the presence of Lp(a), the apo(a) fragment has been reported to bind to fibrin, forming a quaternary complex that promotes a markedly reduced rate of plasminogen activation compared with the classical ternary complex. This interaction was dependent on both KV and the strong lysine binding site (LBS) in KIV type 10, but did not involve the inactive plasminogen-like protease domain [22]. Lp(a) has been shown to compete with both plasminogen and tPA for binding to fibrin, promoting a thrombotic state through preventing plasmin-mediated clot lysis [23, 24] (Figure 1(b)). 

In addition to directly impairing ternary complex formation, Lp(a) can also influence plasminogen activation by associating with inhibitors of each component of the ternary complex. Lp(a) was shown to inhibit the secretion of tPA from EC [25, 26]; however, one other report did not concur [20]. Although all these studies utilised the same endothelial cell source (HUVEC), the lack of effect of Lp(a) on tPA secretion in the latter study may have been due to the absence of serum in their experimental protocol. In this case, a combination of transferrin, selenium, and insulin was used in place of serum, and it may be that cofactors within serum other than these are necessary for this aspect of Lp(a) functionality. Irrespective of an effect on tPA secretion, Lp(a) has been reported to increase expression of plasminogen activator inhibitor-1 (PAI-1, an inhibitor of tPA) in HUVEC and human coronary artery EC (HCAEC) in a protein kinase C (PKC-) dependent mechanism [20, 27]. This was further enhanced by oxidising or glycating Lp(a) [26, 28]. A recent report determined that Lp(a) also associates with other prothrombotic proteins including *α*2-macroglobulin (a plasmin inhibitor) and serine proteinase inhibitor A1 (SERPINA1, a tPA inhibitor) [8]. Therefore, Lp(a) can inhibit activation of plasminogen via multiple pathways—inhibiting the association of plasminogen, fibrin and tPA, reducing availability of tPA, increasing expression of tPA inhibitor PAI-1, and associating with SERPINA1, which taken together can all lead to impairment of fibrinolysis (Figure 1(b)).

### 2.4. Inhibition of TGF*β* Activation

One of the substrates of plasmin is transforming growth factor beta (TGF*β*) [29]. TGF*β* has a variety of cellular effects which can either protect against atherosclerosis (e.g., inhibition of SMC migration [30]) or promote atherosclerosis (e.g., inhibition of EC migration [31] and induction of intercellular adhesion molecule-1 (ICAM-1) expression on EC [32], summarised in Figure 1(a)). In addition, active TGF*β* can reduce transcription of the apo(a) gene [33]. As Lp(a) has been shown to inhibit plasminogen activation, it also prevents activation of TGF*β* leading to an increase in proliferation and migration of cells within vessel walls [34]. This was shown to be dependent on the apo(a) subunit since TGF*β* activation was unaffected by LDL alone [35]. Accordingly, studies using aortic SMC revealed that treatment for 96 h with r-apo(a) did not prevent secretion of latent TGF*β per se*, but did inhibit its activation through a KIV type 9 dependent effect, abrogating the antiproliferative and antimigratory capacity of TGF*β* [36]. A later study in HUVEC revealed that r-apo(a) treatment for 72 h decreased TGF*β* activity as predicted; however, in this case it was accompanied by a 50% decrease in total TGF*β* secreted from the cell suggesting a further mechanism(s) whereby Lp(a) may reduce the bioavailability of TGF*β*. Under those conditions, reduction in TGF*β* activity and production were dependent upon the LBS on KIV type 10 and KV, and on integrin *α*_*v*_ *β*_3_ [37]. 

## 3. Inflammatory Cell Recruitment and Adhesion

**Figure 2:**
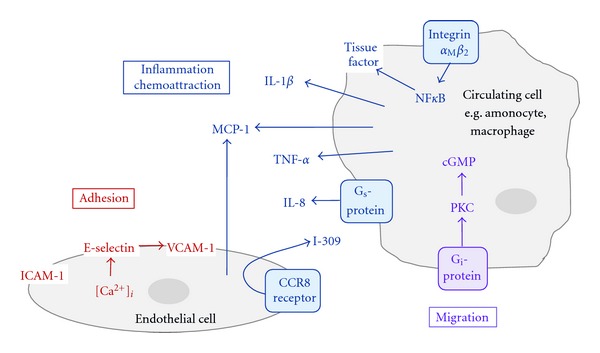
Inflammatory cell attraction and adhesion. Lp(a) encourages homing of inflammatory cells to sites of Lp(a) deposition within the vascular wall. Circulating cells are attracted to the endothelium by inflammatory cytokines induced by Lp(a). They then bind to and migrate through resident EC via adhesion molecules on the endothelial surface, also induced by Lp(a).

One of the main mechanisms through which Lp(a) confers its effects is mediation of inflammation. Plasma Lp(a) levels are reportedly elevated in patients suffering from inflammatory diseases such as Crohn's disease [38] and in the microvasculature of inflammatory lesions in gall bladder, heart, and lymph nodes [20]. Expression of Lp(a) is increased by the proinflammatory cytokine interleukin-6 (IL-6), through binding to multiple sites in the apo(a) promoter [39], prompting speculation that it may act as an acute phase reactant. In addition, Lp(a) has been reported to carry oxidised phospholipids, promote secretion of inflammatory cytokines, attract inflammatory cells to sites of deposition, and encourage transendothelial migration as described below (summarised in Figure 2). 

### 3.1. Transportation of Oxidised Phospholipids

Lp(a) is claimed to be an acute phase reactant, with increased circulating levels being observed following myocardial infarction [40, 41] and percutaneous coronary intervention [42]. It is speculated that this may point to a physiological anti-inflammatory role for Lp(a) in patients with low plasma levels, whereby Lp(a) could bind to and remove oxidised phospholipids from the circulation, preventing further damage. Oxidised phospholipids are proinflammatory in nature and are bound by Lp(a). Although they are often found associated with apoB-100 [43], studies have shown that within the Lp(a) molecule the association was dependent on KV of the apo(a) moiety [44]. The amount of oxidised phospholipid bound to apo(a) remained constant and was unaffected by apo(a) size suggesting that it was bound to apo(a) during synthesis in the hepatocyte and was not derived from plasma LDL [45]. Whilst this may be beneficial in low concentrations, in patients with high plasma levels of Lp(a) preferential binding of oxidised phospholipids may lead to their deposition within the vessel wall, hence promoting atherogenesis [46]. 

### 3.2. Induction of Inflammatory Cytokines

Lp(a) has been shown to induce inflammatory cytokine expression in a cell-type-specific manner. For example, apo(a) induced IL-8 expression in macrophages, but not monocytes. Detailed analysis revealed that Lp(a) induced a 12-fold increase in IL-8 mRNA, whereas apo(a) alone was almost three times more potent in inducing transcription. This was mirrored at the protein level and was dependent on KV and interaction with G_s_ protein receptors. IL-8 induction was not observed by exposure to LDL or Lp(a) moieties without the apo(a) fragment [47] confirming the essential role of the apo(a) moiety in this process. In addition, Lp(a) also induced expression of IL-1*β*, tumour necrosis factor alpha (TNF-*α*), and monocyte chemoattractant protein (MCP-1) in macrophages. Interestingly, pretreatment with 17*β*-estradiol has been shown to attenuate the induction of proinflammatory cytokines and also reduced macrophage migration in an Lp(a) transgenic mouse carotid artery ligation model [48]. Hormone replacement therapy is recognised to modulate plasma Lp(a) levels and cardiovascular risk [49], most likely through an estrogen receptor response element within the promoter of the apo(a) gene [50].

### 3.3. Chemoattraction

Through its functional effects on EC, Lp(a) can induce chemotaxis and attract monocytes via both direct and indirect mechanisms. HUVEC treated with Lp(a) produced MCP-1 [48] and another monocyte chemoattractant, I-309 [51]. I-309 is a CC chemokine commonly secreted by T-lymphocytes and monocytes to attract leukocytes. LDL alone caused a minimal and nonsignificant upregulation of I-309; in further detailed analysis either 17K r-apo(a) (containing all KIV types) or 6K r-apo(a) (containing KIV types 5–10) was investigated. The 6K r-apo(a) fragment was almost twice as effective as 17K r-apo(a) in increasing monocyte chemoattraction. The effect of 6K r-apo(a) was blocked by coincubation with I-309 siRNA or neutralising antibody and occurred through its receptor, CCR8. This is another example that clearly demonstrates the cell-type-specific nature of Lp(a) signalling, as a similar study in neutrophils showed no effect [51]. It is interesting that the smaller r-apo(a) isoform was a more potent monocyte chemoattractant as smaller Lp(a) isoforms are known to be associated with greater CVD risk [52].

Lp(a) has also been shown to induce monocyte chemokinesis directly, independently of the presence of EC. When monocytes were introduced into transwell chamber migration assays, Lp(a) or r-apo(a) both enhanced migration by 400% or 300%, respectively, above basal levels. The magnitude of response observed with Lp(a) suggested that the LDL/apoB-100 subunit may also contribute to this effect. Lp(a) also reportedly activated pertussis-sensitive G_i_-proteins on the cell surface, activated PKC, and increased intracellular levels of cyclic GMP, resulting in chemokinesis. As before, neutrophils exhibited marked resistance to the promigratory effects of Lp(a) [53]. 

In addition to attracting monocytes, Lp(a) may also facilitate their migration though the endothelium. In a study designed to mimic the *in vivo* environment, the membranes of migration chamber inserts were precoated with a confluent layer of HUVEC. Monocytes were loaded into the upper chamber, and the migration assay performed with Lp(a) or r-apo(a) in the lower chamber as chemoattractants. Significantly more monocytes migrated through the EC layer in response to Lp(a) or r-apo(a) than control conditions, in a manner that was dependent upon *α*_M_ *β*_2_ Mac-1 integrin [19]. 

### 3.4. Adhesion of Inflammatory Cells to the Endothelium

In addition to promoting monocyte migration through an endothelial monolayer as described above, Lp(a) has been shown to promote adhesion of monocytes by binding through *α*_M_ *β*_2_ Mac-1 integrin, an effect dependent on the LBS of the apo(a) fragment. Adhesion was enhanced through coincubation with homocysteine, a proatherogenic molecule, yet interestingly pretreatment with aspirin decreased adhesion by 30–40% [19]. Interestingly, aspirin has been reported to lower plasma Lp(a) by reducing transcription of the apo(a) gene [54]. Aspirin may, therefore, modulate the perceived detrimental effects of Lp(a) on multiple levels and may be more beneficial for patients with CVD and high circulating levels of Lp(a) than previously thought.

In addition to binding integrins, Lp(a) has been shown to induce adhesion molecule expression on EC. Treatment of HCAEC with Lp(a) resulted in increased expression of vascular cell adhesion molecule (VCAM)-1 and E-selectin. In this case, apo(a) alone was not sufficient to induce expression and yet removing apo(a) from the Lp(a) molecule also resulted in no effect. It appears that in this case, both components of Lp(a) were essential in mediating the response [55]. Importantly, adhesion molecule expression is highly dependent on the source of EC; HUVEC treatment with Lp(a) or r-apo(a) alone caused a marked increase in ICAM-1 and yet had no effect on VCAM-1 or E-selectin expression [56]. On the other hand, Lp(a) induced expression of all three adhesion molecules (ICAM-1, VCAM-1, and E-selectin) in bovine aortic endothelial cells (BAEC). Intriguingly, in this case pretreatment with 17*β*-estradiol attenuated this effect suggesting an additional mode of cardioprotection by estrogen [48]. It is clear that the source of EC and the experimental model used require careful consideration when interpreting data relating to Lp(a). 

## 4. Vascular Remodelling

**Figure 3:**
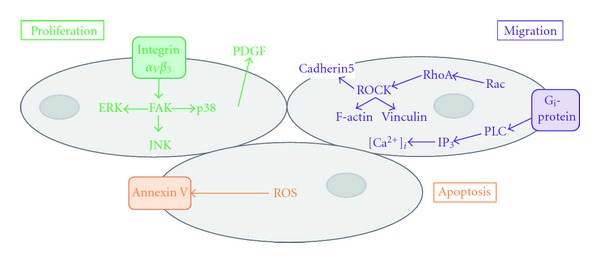
Proliferation and migration of resident vascular wall cells. Lp(a) promotes vascular remodelling by activating multiple signalling pathways within SMC and EC. The response of the cell (e.g., proliferation, migration, or apoptosis) is dependent upon Lp(a) activated signalling cascades that are cell-type specific and dependent upon the oxidation state of the Lp(a) molecule.

The capacity of vessel walls to respond and remodel to external cues is essential for vascular adaptation to physiological processes and also in “pathological” remodelling observed in CVD. Endothelial dysfunction and aberrant proliferation and migration of SMC are characteristic in the development of atherosclerosis [57]. Lp(a) has been observed to be deposited in atherosclerotic lesions [19] and is proposed to mediate vascular remodelling through alterations in the proliferative and migratory capacity of resident EC/SMC cells as described below (summarised in Figure 3). 

### 4.1. Proliferation of Smooth Muscle Cells

Aberrant proliferation of SMC is detrimental to the vessel, and as mentioned earlier Lp(a) has been reported to induce SMC proliferation via inhibition of TGF*β* activation [58]. Treatment of SMC with r-apo(a) for 24–96 h promoted an approximate 60% increase in SMC proliferation, dependent on the LBS in KIV type 9 [36]; however, prolonged exposure to Lp(a) may also promote SMC proliferation via additional apo(a)-independent mechanisms. For example, Lp(a) increased SMC proliferation by ~37% following 5 days of treatment, whereas treatment with LDL alone induced proliferation by 63% prompting the authors to speculate that as well as inhibiting TGF*β* activation, Lp(a) enhanced SMC proliferation through an LDL-dependent pathway [35]. LDL has previously been reported to increase SMC proliferation although this observation is inconsistent and requires further validation [59, 60]. 

Oxidised phospholipids are potent inducers of CVD; the pathogenicity of LDL is greatly increased when the molecule is oxidised [61]. The magnitude of the effect of Lp(a) as an SMC mitogen has been reported to be increased in its oxidised state (oxLp(a)) [62]. A common intracellular signalling pathway linked to proliferation is extracellular signal-related kinase (ERK). Native Lp(a) was seen to activate this pathway and induced SMC proliferation; however, oxLp(a) caused a more robust phosphorylation of ERK and a concomitant increase in SMC proliferation [62]. Therefore, just as the oxidative state of LDL influences its potency as a cardiovascular risk factor, similarly the oxidative state of Lp(a) may also influence its pathogenicity. 

In addition to direct stimulation of SMC proliferation via the apo(a) and LDL moieties and inhibition of TGF*β* activation, Lp(a) may also promote SMC proliferation within the vessel wall by increasing expression of EC-derived platelet-derived growth factor (PDGF), a potent SMC mitogen. Reports have shown that oxLp(a) is capable of inducing PDGF expression in HUVEC [63], which would presumably further enhance SMC proliferation within vessel walls. However, native Lp(a) appears not to induce PDGF expression [20, 63], lending further support to the theory that oxLp(a) is a more potent mediator of vascular dysfunction than native Lp(a). 

### 4.2. Proliferation of Endothelial Cells

Whilst the pathways to Lp(a)-induced SMC proliferation are well defined, the mechanisms of Lp(a) effects on EC appear more complex. Although Lp(a) reportedly induced a 4.5-fold increase in HUVEC proliferation, this was considerably less than the 6-fold induction evident in response to LDL alone suggesting that in this case, the apoB-100/LDL component of Lp(a) may have been more potent in mediating the proliferative response than the apo(a) fragment [64]. Later studies utilising r-apo(a) and Lp(a) in HUVEC observed a comparable increase in proliferation with either treatment, indicating that it was the apo(a) fragment that was responsible for the proliferative response. When EC were treated with apo(a) alone, proliferation was dependent on the LBS in KIV type 10, KV, and integrin *α*_*v*_ *β*_3_. However, when the whole Lp(a) molecule was used, the LBS was no longer an essential requirement for the mitogenic response [37]. Although the first study demonstrated enhanced proliferation with LDL [64], the second reported no effect [37]. These discrepant results may be explained by differences in experimental protocols, in which one study used serum-free culture medium whilst in the other, cells were already stimulated to proliferate with insulin and fibroblast growth factor (FGF) [64]. It seems likely that LDL and growth factors such as insulin may act in a synergistic manner to influence EC proliferation. To add a further level of complexity, Lp(a) has been shown to increase expression of FGF-2 in HUVEC, and the stimulatory effect of Lp(a) was blunted when inhibitors of FGF-2 or G_i_-proteins were included [65]; therefore, synergy between Lp(a) and FGF-2 may also occur. In order to validate such a suggestion, further studies could be performed examining specifically the interplay between apo(a), LDL-apoB-100, and whole Lp(a) in the presence and absence of a range of other putative mitogens. 

Independently of LDL, r-apo(a) has been shown to activate several intracellular signalling cascades in HUVEC that are linked to proliferation, for example, focal adhesion kinase (FAK) and the mitogen-activated kinases (MAPKs) p38, p42/44 ERK and p-54 c-Jun N-terminal kinase (JNK). However, all these phosphorylation events were integrin *α*_*v*_ *β*_3_ dependent [37]. Again, to further clarify the importance of component parts of the Lp(a) molecule, further signalling studies could be performed to address this question.

The mitogenic properties of Lp(a) appear to be consistent between cell types. In mesangial cells, Lp(a) (5 *μ*g/mL) induced a small yet significant enhancement of DNA synthesis that was only evident in serum-free cultures, suggesting that increased proliferation was not due to an inhibitory effect of Lp(a) on TGF*β* activation and was more likely to be dependent upon the apoB-100/LDL fragment [66]. Indeed, increased EC proliferation has been observed with similar concentrations of LDL [67]. Another study in mesangial cells had reported reduced DNA synthesis on exposure to a comparable concentration of Lp(a) [67]; however, this study used low concentrations (0.5%) of serum in the cultures that potentially masked any subtle changes in DNA synthesis. 

In spite of published evidence that Lp(a) is mitogenic, there have been a number of studies suggesting, conversely, that oxLp(a) may promote apoptosis. The reported pro-apoptotic effect of oxLp(a) on HUVEC was mediated as a result of oxidative stress and was counteracted by co-incubation with superoxide dismutase and catalase. Intriguingly, this particular study showed that native Lp(a) also exhibited mild proapoptotic properties; however, this was significantly less so than apoptosis induced by oxLp(a) [68]. A recent report demonstrated that oxLp(a)/apo(a) was able to provoke apoptosis in macrophages that were undergoing endoplasmic reticulum stress. This was dependent on oxidised phospholipids carried by the apo(a) molecule and involved apoptotic signalling via CD36-Toll-like receptor 2 (TLR2) and generation of reactive oxygen species (ROS) [69]. It is interesting that the same concentration of Lp(a) used in two separate studies induced opposing effects of apoptosis [68] and proliferation [64]. It is possible that alternative signalling events occur in EC, whereby Lp(a) stimulates proliferation via the ERK pathway, and apoptosis through ROS generation, depending upon the environment of the EC, thus contributing to dysfunction at multiple levels. 

### 4.3. Migration of Smooth Muscle and Endothelial Cells

In addition to altered proliferation, aberrant migration of SMC and EC also contributes significantly to cardiovascular pathologies. Enhanced migration of SMC treated with r-apo(a) was observed over a period of 24–96 h; the increased speed of SMC migration being dependent on KIV type 9 [36]. Increased migration in HUVEC has also been demonstrated, whereby exposure to r-apo(a) or Lp(a) directly enhanced migration in a scratch wound model. However, in these cells, the promigratory effect was dependent on the LBS in KIV type 10, KV, and integrin *α*_*v*_ *β*_3_. In the case of Lp(a), migration was not prevented by inhibiting the LBS proposing an involvement of the LDL/apoB-100 moiety [37]. However, Lp(a) was more potent than LDL in promoting HUVEC migration suggesting that it is the apo(a) fragment playing the major role with minimal contribution from LDL/apoB-100 [65].

The molecular mechanisms that underlie cellular migration are complex but result in coordinated rearrangement of the actin cytoskeleton and increased formation of f-actin stress fibres to promote cell motility. Accordingly, apo(a) has been reported to influence stress fibre formation. When either HUVECs or HCAECs were incubated with concentrations of r-apo(a) as low as 25 nM, there was a notable increase in the appearance of stress fibres, an effect reproduced by Lp(a). However, incubation with plasminogen or LDL had no effect, demonstrating the apo(a)-dependency of stress fibre formation. The RhoA/Rho kinase (ROCK) pathway is a key modulator of f-actin cytoskeleton arrangement [71], and its central role in mediating the effect of apo(a) was demonstrated by abolition with ROCK inhibitors [72]. 

In addition to increasing stress fibre formation, r-apo(a) has been reported to increase endothelial permeability by inducing cell contraction [70], an effect that would facilitate transendothelial migration of monocytes as previously observed *in vitro* [19]. Detailed examination of the underlying mechanism in HUVEC revealed that contraction was dependent on the strong LBS within KIV type 10 [72], enhancing stress fibre formation and cell contraction through phosphorylation of myosin light chain (MLC) via two mechanisms. Firstly, by increasing MLC kinase (MLCK) activity, and secondly by increasing myosin phosphatase target subunit 1 (MYPT1) activity (an inhibitor of MLC phosphatase) thereby promoting phosphorylation and activity of MLC [70]. Apo(a) has been shown to activate RhoA in HUVEC [72] and accordingly phosphorylation of MLC was dependent on the activation of RhoA and ROCK [70]. 

Alterations in intracellular calcium levels ([Ca^2+^]_i_) are known to have a significant impact on many cellular functions including migration [73]. However, the influence of r-apo(a) on [Ca^2+^]_i_ is controversial and is reported to vary according to cell type and species. For example, one report demonstrated that treatment of HUVECs with r-apo(a) did not alter Ca^2+^ homeostasis; however, some of the recognised effects of r-apo(a) were mediated via Ca^2+^-sensitive proteins such as MLCK [72]. Intriguingly, another report revealed that when a variety of human EC (coronary artery, aortic, or heart microvascular) were treated with Lp(a), a proportion of cells within any cell population responded whilst others did not [55]. Furthermore, treatment of human mesangial cells with Lp(a) caused a transient increase in [Ca^2+^]_i_ that was dependent on pertussis-sensitive G_i_ proteins [66]. Clearly, the mechanisms via which Lp(a) may or may not affect Ca^2+^-dependent cellular effects require extensive further study before any firm interpretations can be drawn.

## 5. Conclusions

In this paper, we have evaluated published evidence that supports a role of Lp(a) as a proatherogenic CVD risk factor. It is clear that Lp(a) is capable of a broad spectrum of functional effects within the cardiovascular system, through both its LDL/apoB-100 moiety and apo(a) chain (Table 1). Such effects are potentially detrimental to cardiovascular health, contributing to CVD progression via activation of a diverse range of cell surface receptors and signalling pathways, principally:

1. modulation of platelet aggregation (*α*_IIb_ *β*_3_),
2. reduction in fibrinolysis (*α*_M_ *β*_2_, *α*_*v*_ *β*_3_, NF*κ*B, PKC),
3. recruitment of inflammatory cells (*α*_M_ *β*_2_, E-selectin, ICAM-1, VCAM-1, IL-1*β*, IL-8, PKC),
4. induction of vascular remodelling (*α*_*v*_ *β*_3_, Ca^2+^, MAPK, RhoA, ROS).

A cognate receptor for Lp(a) remains elusive and indeed may not exist. However, the capacity of Lp(a) to activate a diverse range of proteins and receptors suggests that it is unlikely to activate a single receptor that could yield a single therapeutic target. A combination of therapies may, therefore, offer alternative benefits in reducing cardiovascular damage induced by Lp(a). For example, treatment with aspirin to reduce apo(a) gene expression in addition to using statins to inhibit RhoA-dependent functional effects on EC and SMC may be of value. Indeed, improved bypass patency rates in patients with high plasma Lp(a) receiving statin therapy compared to those not taking statins have been reported [74]. Over recent decades robust cholesterol management through statin therapy has had a profound effect on cardiovascular protection, although a similar management for plasma Lp(a) seems unlikely at present. Dissecting the signalling mechanisms that Lp(a) exploits in cardiovascular cells is therefore key to identifying suitable targets for therapeutic intervention in the short term. 

## Figures and Tables

**Table 1:** Lp(a) subunits responsible for cellular effects.

Process	LDL-apoB-100	Apo(a)	Region	References
Thrombosis				
(i) Platelet aggregation	√	√		[11–17]
(ii) ↑ tissue factor pathway	—	√		[19–21]
(iii) ↓ plasminogen activation	—	√	KIV type 10, KV	[8, 20, 23–28]
(iv) ↓ TGF*β* activation	×	√	KIV type 9	[34–37]
(v) ↓ TGF*β* production	—	√	KIV type 10, KV	[37]
Inflammation				
(i) Oxidised phospholipids	×	√	KV	[44–46]
(ii) ↑ monocyte migration	√	√		[19, 51, 53]
(iii) ↑ monocyte adhesion	×	√	KIV type 10	[19]
Vascular remodelling				
(i) ↑ SMC proliferation	√	√	KIV type 9	[35, 36, 58, 62]
(ii) ↑ SMC migration	—	√	KIV type 9	[36]
(iii) ↑ EC proliferation	√	√	KIV type 10, KV	[37, 64, 65]
(iv) ↑ EC migration	√	√	KIV type 10, KV	[37, 65]
(v) ↑ EC contraction	—	√	KIV type 10	[70]

For detailed information, see main body of text and highlighted references. Key: √ = positive for effect, × = negative for effect, — = not tested.
